# Risk Factor Analysis in Vascular Access Complications for Hemodialysis Patients

**DOI:** 10.3390/diagnostics15010088

**Published:** 2025-01-02

**Authors:** Cristian Dan Roşu, Sorin Lucian Bolintineanu, Bogdan Florin Căpăstraru, Roxana Iacob, Emil Robert Stoicescu, Claudia Elena Petrea

**Affiliations:** 1 1st Surgery Clinic, “Victor Babes” University of Medicine and Pharmacy Timisoara, Eftimie Murgu Square No. 2, 300041 Timisoara, Romania; rosu.cristian@umft.ro; 2 Department of Anatomy and Embriology, “Victor Babes” University of Medicine and Pharmacy Timisoara, 300041 Timisoara, Romania; roxana.iacob@umft.ro (R.I.); claudia.petrea@umft.ro (C.E.P.); 3 Doctoral School, “Victor Babes” University of Medicine and Pharmacy Timisoara, Eftimie Murgu Square No. 2, 300041 Timisoara, Romania; bogdan.capastraru@umft.ro; 4 Research Center for Medical Communication, “Victor Babes” University of Medicine and Pharmacy Timisoara, Eftimie Murgu Square No. 2, 300041 Timisoara, Romania; 5 Field of Applied Engineering Sciences, Specialization Statistical Methods and Techniques in Health and Clinical Research, Faculty of Mechanics, “Politehnica” University Timisoara, Mihai Viteazul Boulevard No. 1, 300222 Timisoara, Romania; stoicescu.emil@umft.ro; 6 Radiology and Medical Imaging University Clinic, “Victor Babes” University of Medicine and Pharmacy Timisoara, Eftimie Murgu Square No. 2, 300041 Timisoara, Romania; 7 Research Center for Pharmaco-Toxicological Evaluations, “Victor Babes” University of Medicine and Pharmacy Timisoara, Eftimie Murgu Square No. 2, 300041 Timisoara, Romania

**Keywords:** chronic kidney disease, hemodialysis, vascular access complications, thrombosis, hemorrhage, non-maturation, aneurysmal dilation, venous hypertension

## Abstract

**Background:** Chronic kidney disease (CKD) and renal failure remain critical global health challenges, with vascular access complications posing significant obstacles in hemodialysis management. **Methods:** This study investigates the early and late complications associated with vascular access procedures in a cohort of 1334 patients from Timiș County Emergency Clinical Hospital. Patients were categorized into early complications, occurring within 30 days postoperatively, and late complications, developing beyond this period. Demographic data, comorbidities, and lifestyle factors, including age, gender, body mass index (BMI), smoking status, hypertension, diabetes, and cardiovascular disease (CVD), were recorded and analyzed. Early complications included thrombosis, hemorrhage, edema, and non-maturation, while late complications involved thrombosis, aneurysmal dilation, venous hypertension, and infections. **Results**: Hemorrhage (32.3%) and thrombosis (30.8%) were the most prevalent early complications, influenced significantly by diabetes and hypertension. Non-maturation showed a strong association with diabetes and cardiovascular disease (odds ratio: 1.70). For late complications, thrombosis was most frequent, with hypertensive patients exhibiting increased risk (relative risk: 1.18). BMI was a significant factor in both early and late complications. Risk analysis using odds ratios and relative risks revealed distinct patterns of complication risks based on comorbidities and smoking status. Logistic regression modeling for thrombosis demonstrated moderate predictive accuracy (AUC: 0.64). **Conclusions**: These findings suggest that clinical interventions, such as stricter perioperative glycemic and blood pressure control, and personalized surgical strategies for patients with high BMI or comorbidities, could significantly reduce the incidence of vascular access complications and improve outcomes in this high-risk population.

## 1. Introduction

Chronic kidney disease (CKD) and renal failure are significant global health concerns, with CKD affecting approximately 10–15% of the global population. The prevalence varies across regions, with higher rates reported in low- and middle-income countries due to limited access to healthcare and higher rates of risk factors such as diabetes mellitus and arterial hypertension. CKD is often asymptomatic in its early stages, leading to delayed diagnosis and progression to end-stage renal disease, which requires dialysis or kidney transplantation. Epidemiologically, CKD disproportionately affects older adults, and its incidence is rising due to aging populations and the increasing burden of chronic diseases [1]. Although there is increasing awareness of the significance of CKD in health care, kidney failure persists as a major global public health challenge. Worldwide, the prevalence of kidney failure is steadily climbing, contributing to 1.2 million deaths in 2015 [2].

Hemodialysis is the primary alternative to kidney transplantation, with approximately 4 million patients worldwide [3]. Vascular access remains a major challenge for those on chronic hemodialysis, requiring innovative solutions to ensure long-term access. Options include tunneled dialysis catheters, prosthetic grafts, and arteriovenous fistulas (AVFs) [4]. Compared to grafts and catheters, AVFs offer higher patency, lower infection risk, greater durability, and fewer interventions. However, AVFs have a significant non-maturation rate (20–50%), leading many patients to switch to tunneled catheters within six months [4,5]. To address these issues, innovations like external support devices, advanced imaging, and minimally invasive techniques are being explored. The Dialysis Access Consortium Fistula Trial’s high maturation failure rates led to the formation of the Hemodialysis Fistula Maturation Consortium to investigate factors influencing AVF maturation [6].

AVFs, although considered the preferred vascular access for hemodialysis, are still prone to a range of complications. Stenosis and thrombosis are among the most common issues, with stenosis often occurring near the anastomosis site, leading to restricted blood flow [6,7]. Other complications include infections, which, although less frequent than in other types of access, can still pose serious risks, particularly in immunocompromised patients [8]. Aneurysmal dilation is another concern, occurring when the vein wall weakens and dilates, potentially leading to rupture if not properly managed [9,10]. Additionally, complications such as bleeding or hematoma formation can arise, particularly during cannulation for dialysis sessions [6].

The risk of these complications is influenced by several patient-specific factors. Age is a significant factor: older patients often have weak vessels which may be more susceptible to stenosis and aneurysm formation. Diabetes mellitus and arterial hypertension, two common conditions among hemodialysis patients, can adversely affect vascular health, increasing the risk of both stenosis and thrombosis. Cardiovascular disease and congestive heart failure can also impact AVF patency, as reduced cardiac function may lead to insufficient blood flow to support a healthy AVF. Other factors, such as the quality of preoperative vascular mapping, cannulation technique, and even the specific site chosen for AVF placement, further influence the risk of complications [11,12,13,14].

With the rising prevalence of CKD and kidney failure, the substantial morbidity, mortality, and expense associated with dialysis vascular access, and the likelihood that hemodialysis will continue as the primary kidney replacement therapy in the near future, there is a pressing clinical need to overcome current challenges in vascular access for hemodialysis. This need is fueling essential innovation and research into new techniques and care processes aimed at enhancing long-term vascular access for patients on chronic hemodialysis.

## 2. Materials and Methods

### 2.1. Data Collection and Datasets

This study focused on patients admitted to the Timiș County Emergency Clinical Hospital to investigate the occurrence of early and late complications following vascular access procedures. Prior to undergoing surgery, all patients underwent a thorough evaluation, during which demographic data and comorbidities were recorded. Key variables included patient age, gender, smoking status, and body mass index (BMI); and the presence of pre-existing conditions, such as arterial hypertension, diabetes mellitus, and cardiovascular disease (CVD).

Patients were then categorized into two datasets based on when complications were observed. The first dataset comprised patients who experienced complications within the immediate postoperative period (less than 30 days). The second dataset focused on patients who developed complications over a more extended postoperative period, reflecting long-term challenges in maintaining vascular access functionality. Both datasets included categorical health conditions recorded as either “yes” or ”no”. Formatting adjustments were made to ensure consistent data representation across analyses. Cases with missing data were excluded from the analysis to ensure consistency across variables.

### 2.2. Data Preprocessing

In preparing the data, continuous variables (age, BMI) were standardized to facilitate accurate modeling, while categorical variables (gender, smoking status, hypertension, diabetes, and CVD) were encoded as binary indicators (male/female for gender and yes/no for comorbidities). Since both datasets were complete, no imputation was necessary. Each complication was firstly categorized as either early or late based on its onset following the medical intervention (less than 30 days/more than 30 days).

### 2.3. Statistical Analysis

Descriptive statistics were used to summarize the demographic characteristics and complications. Continuous variables were expressed as means ± standard deviations (SDs), and categorical variables were presented as percentages. The distribution of complications across demographic factors (age, gender, smoking status, and comorbidities) was examined. A univariate analysis was performed to assess the relationships between demographic and clinical factors (age, gender, BMI, smoking status, and comorbidities) and the likelihood of developing various complications (early and late). The odds ratios (ORs) with 95% confidence intervals (CIs) were calculated for the association between age, gender, BMI, smoking status, and comorbidities with each complication. To identify independent predictors of complications, logistic regression was applied to assess the relationships between demographic and clinical factors and the occurrence of early and late complications. Due to the smaller sample sizes in some complications, logistic regression models were selectively applied to complications with sufficient cases. For thrombosis, logistic regression assessed the influence of age, gender, BMI, smoking status, hypertension, diabetes, and CVD on complication risk. Model performance was evaluated with metrics such as ROC AUC, and accuracy, and feature importance was derived from model coefficients. All statistical analyses were performed using SPSS version 23.0 (IBM Corp, Armonk, NY, USA). A *p*-value of < 0.05 was considered statistically significant.

### 2.4. Ethical Consideration

This study was conducted in accordance with the principles outlined in the Declaration of Helsinki and was approved by the Ethics Committee of the Timiș County Emergency Clinical Hospital (501/15.11.2024). All patients included in the study provided written informed consent prior to data collection, ensuring their voluntary participation. Patient confidentiality was strictly maintained throughout the study, with all data anonymized and stored securely to prevent unauthorized access.

## 3. Results

**Figure 1:**
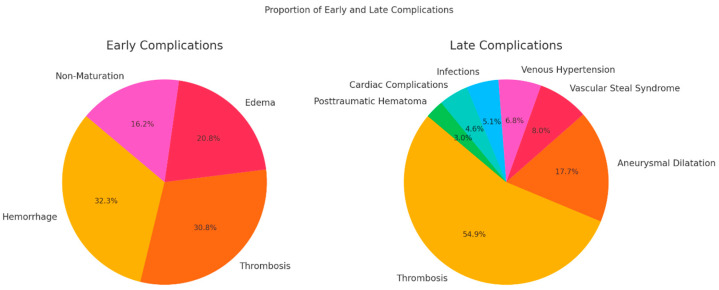
Prevalence of early and late complications in the entire cohort of patients.

A total of 1334 patients were included in the study, with 130 patients experiencing early complications and 237 patients presenting with late complications following medical interventions. Early complications were categorized as hemorrhage, thrombosis, edema, and non-maturation, occurring within the immediate postoperative period. Late complications, which developed over an extended postoperative period, included thrombosis, aneurysmal dilation, vascular steal syndrome, venous hypertension, infections, cardiac complications, and posttraumatic hematoma (Figure 1).

### 3.1. Early Complications

In this cohort of 130 patients, the average age is 66.2 years, and the mean BMI is 26.9 kg/m^2^. The gender distribution shows 62.3% male and 37.7% female patients. Regarding smoking status, 55.4% have never smoked, 28.5% are former smokers, and 16.2% are current smokers (Table 1).

The analysis of complication rates across demographic factors reveals distinct patterns based on gender, age, and smoking status. Among female patients, edema and hemorrhage each occur in 26.5% of cases, followed by non-maturation at 16.3% and thrombosis at 30.6%. In contrast, male patients experience hemorrhage most frequently (35.8%), followed closely by thrombosis (30.9%). Edema affects 17.3% of male patients, while non-maturation occurs in 16.0%.

Patients under 50 show relatively high rates of edema and hemorrhage (33.3% each), with thrombosis at 22.2% and non-maturation at 11.1%. In the 50–60 age group, non-maturation is the most common complication, affecting 36.0%, followed by thrombosis (28.0%) and edema (24.0%). For patients aged 60–70, hemorrhage and thrombosis are the leading complications, each affecting 36.7% of patients, while edema and non-maturation occur less frequently at 10.0% and 16.7%, respectively. Among patients over 70, hemorrhage is the most prevalent complication at 38.6%, followed by thrombosis (31.6%), edema (21.1%), and non-maturation (8.8%).

Smoking status also influences complication rates. Current smokers exhibit higher rates of edema and thrombosis, each affecting 33.3%, with hemorrhage occurring in 33.3% of cases and non-maturation at 4.8%. Former smokers experience thrombosis most frequently (37.8%), followed by hemorrhage (29.7%), edema (21.6%), and non-maturation (10.8%). Among patients who have never smoked, hemorrhage is the most common complication (33.3%), followed by thrombosis (26.4%), non-maturation (22.2%), and edema (18.1%).

Hemorrhage was the most prevalent complication, affecting 32.3% of the cohort, followed closely by thrombosis at 30.8%. Edema and non-maturation were less common, occurring in 20.8% and 16.2% of the patients, respectively.

Within each complication type, the prevalence of comorbidities varied. Among patients with edema, 55.6% had hypertension, 25.9% had diabetes, and 33.3% had CVD. In the hemorrhage group, 38.1% of patients had hypertension, 45.2% had diabetes, and 35.7% had CVD. Non-maturation complications showed a higher prevalence of comorbidities, with 47.6% of patients experiencing hypertension, 47.6% having diabetes, and 47.6% affected by CVD. In the thrombosis group, 52.5% of patients had hypertension, 30.0% had diabetes, and 35.0% had CVD.

Our analysis of comorbidities across the cohort revealed that 39.2% of patients had two or more conditions among hypertension, diabetes, and CVD. Our analysis of complication rates based on underlying health conditions revealed notable differences between patients with and without hypertension, diabetes, and CVD. Among patients without hypertension, hemorrhage was the most common complication, affecting 38.2% of this group, followed by thrombosis at 27.9%, edema at 17.6%, and non-maturation at 16.2%. In contrast, hypertensive patients had a slightly different pattern, with thrombosis remaining prevalent (33.9%) and hemorrhage occurring in 25.8% of cases. Edema and non-maturation were more common in hypertensive patients, at 24.2% and 16.1%, respectively.

For patients without diabetes, thrombosis was the most frequent complication, affecting 34.1%, followed by hemorrhage (28.0%), edema (24.4%), and non-maturation (13.4%). Among diabetic patients, hemorrhage was more common, occurring in 39.6% of cases, while non-maturation occurred in 20.8%, suggesting an association between diabetes and non-maturation. Thrombosis and edema rates were somewhat lower among diabetic patients, at 25.0% and 14.6%, respectively.

The presence of CVD also influenced complication patterns. Among patients without CVD, hemorrhage was the most common complication, affecting 32.9%, followed by thrombosis (31.7%), edema (22.0%), and non-maturation (13.4%). In patients with CVD, hemorrhage remained common at 31.3%, with thrombosis at 29.2%, non-maturation at 20.8%, and edema at 18.8%.

#### Risk Analysis

A univariate analysis was conducted to assess the relationship between age, gender, BMI, comorbidities, and smoking status and the likelihood of experiencing various early complications (Table 2). Gender was associated with an increased risk of thrombosis and non-maturation, but not with hemorrhage. Thrombosis shows a slightly increased likelihood in hypertensive patients (OR = 1.32) and a lower likelihood in diabetic patients (OR = 0.65) and those with CVD (OR = 0.90). Hemorrhage appears notably influenced by diabetes, with an odds ratio of 1.67, indicating a higher risk among diabetic patients. Conversely, hypertension is associated with a reduced risk of hemorrhage (OR = 0.57), while CVD shows minimal impact (OR = 0.93). Edema shows an increased likelihood in hypertensive patients (OR = 1.47), suggesting higher risk in this group, while diabetic patients have a lower likelihood of developing edema (OR = 0.55). Patients with CVD show a slightly reduced risk of edema (OR = 0.84). For non-maturation, both diabetes and CVD show an increased likelihood (OR = 1.70 for each), indicating that patients with these comorbidities may be at higher risk. Hypertension presents a slight association with non-maturation (OR = 1.10).

### 3.2. Late Complications

Among the 237 patients with late complications, the mean age was 62.4 years, and the mean BMI was 30.3 kg/m^2^. The gender distribution showed a slight predominance of females, with 53.6%, while males accounted for 46.4% (Table 3).

In patients under 50, venous hypertension and posttraumatic hematoma each occur in about 33.3% of cases, while thrombosis is also notable, affecting around 22.2%. Non-maturation is less frequent here, showing up in only about 11.1% of cases.

For those aged 50–59, aneurysmal dilation stands out as the most common complication, affecting 36.0% of patients, followed by thrombosis at 28.0%. Venous hypertension appears in around 24.0% of cases.

Patients in their 60s most frequently experience thrombosis and infections, each affecting about 36.7%. Venous hypertension and aneurysmal dilation are less common in this group, appearing at 16.7% and 10.0%, respectively, suggesting an increased risk for infections and clotting issues as patients age.

In the 70+ group, thrombosis is the leading complication, impacting 38.6%, and cardiac complications affect around 31.6%. Venous hypertension and posttraumatic hematoma show up in about 21.1% of cases, while aneurysmal dilation becomes rarer, at 8.8%.

Among current smokers, aneurysmal dilation is the most common complication, affecting about 33%, followed by thrombosis at 28%. Venous hypertension and infections are somewhat less frequent in this group, with each affecting around 15%. For former smokers, thrombosis is the leading complication, impacting around 40%. In the non-smokers group, venous hypertension is slightly more common, affecting around 30%, with infections appearing in roughly 20%. Thrombosis is less frequent here, affecting about 15% of individuals.

In patients with hypertension, thrombosis is notably high, affecting around 45%, while venous hypertension and cardiac complications follow, affecting 30% and 25%, respectively. Among those with diabetes, infections are more frequent, affecting around 35%, with thrombosis also significant at about 30%. Venous hypertension impacts roughly 20%. For patients with CVD, thrombosis affects about 50%, with cardiac complications following closely at 40%. Lastly, venous hypertension appears in roughly 20% of individuals.

#### 3.2.1. Risk Analysis

A univariate analysis was conducted to assess the relationship between age, gender, BMI, comorbidities, and smoking status and the likelihood of experiencing various late complications (Table 4). The analysis shows that smoking status has varied effects on the risk of developing thrombosis. For those who were never smokers, the relative risk of thrombosis is slightly elevated at 1.10, but this association lacks strong statistical significance. Current smokers appear to have a slightly lower risk of thrombosis (RR = 0.83), suggesting a weaker link between smoking and thrombosis in this dataset. For former smokers, the relative risk is close to neutral, at 1.07.

Regarding comorbidities, hypertension is linked to a moderate increase in thrombosis risk, with an odds ratio of 1.42. This suggests that hypertensive patients may have an elevated risk for thrombosis, though the statistical significance is modest. Similarly, diabetes shows an odds ratio of 1.63, indicating a potential but not definitively significant increase in risk for diabetic patients. Cardiovascular disease also shows an association with thrombosis.

Patients with hypertension had an increased likelihood of aneurysmal dilatation, with an odds ratio of 1.80, indicating a stronger association. Diabetes and cardiovascular disease also contributed to slightly elevated risks for aneurysmal dilatation, with odds ratios of 1.13 and 1.20. In contrast, hypertension and diabetes appeared to lower the risk of infections, with odds ratios of 0.61 and 0.88.

#### 3.2.2. Logistic Model

In this dataset, thrombosis stands out as the only complication for which a logistic regression model can provide reasonably reliable predictions. The dataset contains 130 cases of thrombosis out of 237 patients, giving the model enough positive cases to detect meaningful relationships between thrombosis and predictors such as age, gender, smoking status, BMI, hypertension, diabetes, and CVD.

The logistic regression model for thrombosis achieved an accuracy of 64.6% and an ROC AUC of 0.64, indicating moderate ability to distinguish between patients with and without thrombosis. The model’s precision was 73%. With a recall of 65.5%, the model was able to correctly identify 65.5% of actual thrombosis cases. These metrics show that the model can reasonably balance true-positive and false-positive predictions for thrombosis, making it a useful tool for identifying patients at higher risk of this complication.

For other complications, such as aneurysmal dilation, infections, vascular steal syndrome, and cardiac complications, logistic regression was less effective due to the small number of cases for each of these outcomes. With fewer than 50 cases for each of these complications (and as few as 11 for cardiac complications), the model struggled to identify patterns and showed low predictive power, with ROC AUC scores near 0.5 and zero precision and recall in many cases.

**Figure 2:**
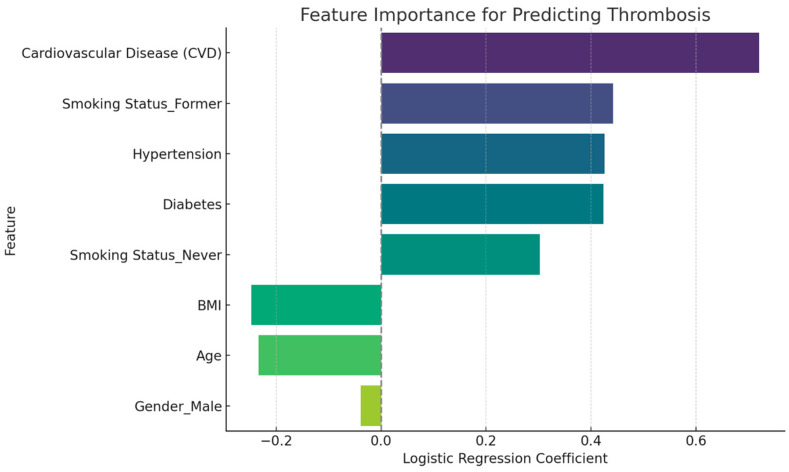
Logistic regression feature importance for predicting thrombosis. Positive coefficients indicate features associated with an increased likelihood of thrombosis, while negative coefficients suggest a reduced likelihood.

Given these limitations, thrombosis was the only complication suitable for logistic regression modeling, as it had a sufficient number of cases and showed moderately reliable predictive performance (Figure 2).

## 4. Discussion

AVFs are the gold standard for hemodialysis vascular access due to their superior patency and lower complication rates compared to grafts and catheters. However, up to 43% of AVFs fail to mature or develop thrombosis [14]. Factors contributing to maturation failure include age, vessel diameter, blood flow, and comorbidities like diabetes and cardiovascular disease [15]. In this study, we analyzed the prevalence and risk factors for early and late complications following AVF creation. The most common early complications were hemorrhage and thrombosis, while late complications included thrombosis, aneurysmal dilation, and venous hypertension. Age, gender, smoking status, and comorbidities such as hypertension and diabetes were found to influence complication rates. Patients with hypertension had a higher likelihood of thrombosis, while diabetes was associated with a higher risk of hemorrhage and non-maturation.

While technical errors can be reduced with careful technique [16], stenoses (78%) and accessory veins (46%) are primary causes of early failure [17]. A meta-analysis found that only 26% of AVFs were fully mature by 6 months, with older patients experiencing longer maturation times and lower success rates [9]. Comorbidities such as CVD, peripheral arterial disease, diabetes mellitus, and diminished physical activity were linked to slower maturation, but arterial hypertension had a minor benefit on maturation levels. However, the Hemodialysis Fistula Maturation Study recently reported fewer early thrombosis cases in diabetes mellitus patients, suggesting that better preoperative planning and screening could enhance outcomes in this group [15]. The optimal hemodialysis approach ensures steady long-term flow, low complication rates, and minimal need for interventions. Non-maturation remains a common AVF complication [18]. New techniques, such as external support devices, demonstrate high unassisted maturation rates (80% at 1 month, 79% at 3 months, and 74% at 6 months) and primary patency rates up to 95%, surpassing traditional methods where patency ranges from 40 to 60%, and 15–40% require revisions [18]. Early clinical and ultrasound evaluations, particularly within six weeks, are essential for timely intervention. Additionally, heart conditions, such as reduced ejection fraction, can negatively affect secondary patency rates [19].

Obesity, defined as a BMI over 30, is increasingly common among kidney failure patients [20] and is associated with impaired AVF maturation [21,22]. Obese patients have lower intra-operative blood flow compared to non-obese patients [23], and while obesity is linked to higher survival rates in hemodialysis, normal or low BMI correlates with worse outcomes [24]. High muscle mass and waist circumference are associated with reduced mortality risk in hemodialysis patients [24,25]. Obese patients often have similar AVF patency rates as non-obese individuals, but those with extremely high BMI may experience poorer long-term vascular access survival [24,26]. Decreased durability may be due to the constriction of the outflow vein by subcutaneous tissue, complicating cannulation. Interestingly, a prospective cohort study involving 197 patients found that diabetes alone was not a significant factor in primary AVF failure. However, diabetic patients using three or more antihypertensive medications were identified as being at higher risk for failure [27]. A meta-analysis of 28 studies found that AVF failure is linked to elevated total cholesterol and LDL levels, suggesting a role for lipid-lowering therapy in preventing AVF failure [13].

CVD is a significant concern in patients with end-stage renal disease, particularly those undergoing hemodialysis with AVFs. In a cohort of patients who underwent kidney transplantation, 12.5% experienced AVF-related complications requiring intervention, with 16.2% related to cardiac decompensation and distal arm hypoperfusion [28]. While AVF closure has shown some benefits in transplant allograft survival, it has not been consistently linked to improved outcomes in all studies [29]. AVFs are associated with high-output heart failure and can worsen existing heart conditions or contribute to new-onset congestive heart failure. Although the incidence is low, some cases of AVF-related worsening CHF have been reported, even years after AVF creation [29]. AVFs also exacerbate left ventricular hypertrophy; however, closure can lead to regression of LVH [30]. Additionally, AVF presence is an independent risk factor for pulmonary hypertension (PH), with prevalence rates ranging from 12% to 45% in hemodialysis patients [31,32], further emphasizing the need for cardiovascular monitoring in these patients.

Diabetes is a significant risk factor for early complications in AVF creation. In the study of Bahrami-Ahmadi et al., the history of diabetes was not significantly different between the early failure group and the control group (23.31% vs. 41.1%, *p* = 0.153), and there were no notable differences in diabetes duration or HbA1c levels. However, hypertension was significantly less prevalent in the early failure group compared to the control group (46.7% vs. 73.2%), with logistic regression analysis showing a trend toward statistical significance (OR, −2.67; 95% CI, −0.97 to −7.36, *p* = 0.061) [33]. Other studies have reported higher AVF failure rates and mortality in diabetic patients undergoing hemodialysis [34]. A systematic review also highlighted an increased risk of AVF failure in diabetic end-stage renal disease patients [35]. Although diabetes itself may not directly cause AVF failure, it affects vascular healing and can worsen outcomes, increasing the likelihood of early complications. Additionally, poor glycemic control and related comorbidities further impair AVF maturation, making diabetes an important risk factor for AVF failure [35]. In our study, among patients with early complications, hypertension was significantly more prevalent in the non-failure group compared to the early failure group (73.2% vs. 46.7%, *p* = 0.002). The prevalence of diabetes was higher in the non-failure group (41.1%) compared to the early failure group (23.3%); however, this difference was not statistically significant (*p* = 0.153).

One study has showed that stenosis was the most prevalent complication (34.3%). Thrombosis was also a significant complication, observed in 5.1% of patients, while other complications included aneurysm (0.6%), infection (0.3% before), and bleeding or hematoma (0.3%) [6]. In another study, out of 374 hemodialysis patients, comorbidities were documented in 309 (83.6%) cases. Among these patients, 48% had diabetes mellitus, 30% had ischemic heart disease, 26% had peripheral vascular disease, 25% had congestive heart failure, and 16% had cerebrovascular disease [36]. An analysis of 13 studies, including over 300,000 patients, demonstrated a strong correlation between obesity and delayed AVF maturation, decreased primary patency rates, and a greater requirement for reintervention [37]. In our study, the most prevalent early complication was hemorrhage (32.3%), while thrombosis (45%) was the most common late complication.

Out of 188 patients, the study of Lucas et al. revealed 111 vascular access complications in 83 patients (44%), with stenosis and thrombosis being the most common issues. A procedure was required to address the first complication in 66 patients (79%), most frequently angioplasty (59%), followed by surgical repair (30%), thrombectomy (10%), and fistula closure (1%). In 17 patients, the fistula was deemed permanently failed. Treatment was successful in 75% of cases, with no significant difference in success rates across groups [38].

A retrospective study of 277 patients by Li et al. found that female gender was linked to lower AVF maturation rates, likely due to narrower vessel diameters and reduced venous dilation in women [39]. Nguyen B et al. reported that age is linked to primary AVF failure in a long-term study involving 100 patients [40]. Similarly, Zi-Ming Wan et al. found that, with every additional 20 years of age, the risk of maturation impairment increased by over 50%, likely due to vessel thickening and damage from conditions such as diabetes mellitus and peripheral vascular disease [11]. However, other large-sized retrospective studies reported no significant gender differences in AVF maturation rates [12,41,42]. In our study, among early complications, hemorrhage and thrombosis were more frequent in males, while edema was more prevalent in females. Complication rates also varied by age, with patients under 50 experiencing higher rates of edema and hemorrhage (33.3%), whereas thrombosis was most common among those over 70 (38.6%).

Research indicates that both elevated and low blood pressure levels impact AVF maturation. Lower pre- and postoperative pressures are associated with poor outcomes, while a postoperative systolic pressure of 120–139 mmHg significantly decreases the risk of fistula failure compared to pressures below 119 mmHg [43].

Thrombosis is the predominant late complication, encountered in 130 AVFs (55.9%), with higher occurrence in distal fistulas (56.2%) compared to proximal ones (43.8%). Like early thrombosis, late thrombosis often coincides with stenosis; however, limited imaging availability has hindered precise assessment. Patients with conditions associated with hypercoagulability may have an increased risk of developing thrombosis due to their predisposition to abnormal clot formation [44].

Stenoses occurring in early periods are most commonly juxta-anastomotic, while those arising later are typically located within 4 cm of the anastomosis [45] Additionally, aneurysmal dilation has been reported in around 10% of cases [46], and an estimated 80% of vascular access dysfunctions are related to thrombosis.

AVFs may develop true aneurysms and pseudoaneurysms, which occur due to damage in the vessel wall, creating a contained hematoma. Although definitions for an AVF aneurysm differ, rates of aneurysm formation in fistulas are reported to be between 43% and 60%. Many of these vascular access aneurysms are stable and asymptomatic, allowing patients to continue dialysis without issues, despite noticeable enlargement. However, some aneurysms enlarge and risk rupture, with rupture rates reported to be between 0.8% and 5.6%, often requiring urgent intervention through revision or ligation [9].

Aneurysmal dilation, often resulting from post-stenotic dilation, affected 42 AVFs (17.7%) in our cohort, with a higher prevalence in proximal locations (81%) compared to distal ones (19%). These findings align with a study by Murphy et al. [10], who reported a 6.38% incidence of aneurysms. Aneurysmal dilations frequently develop in the post-anastomotic segment due to stenosis or repetitive puncturing when puncture rotation protocols are not followed. Cora et al. reported a case of a large extracranial carotid artery pseudoaneurysm following improper placement and removal of a temporary hemodialysis catheter [47].

Vascular access thrombosis is a frequent issue for hemodialysis patients, often resulting in missed dialysis sessions, catheter insertions, and even abandonment of grafts or fistulas. Studies have also explored the association between thrombosis and other medical conditions that may increase the likelihood of this complication [7].

Infectious complications are a significant concern in vascular access, being the second leading cause of fistula non-functionality and the primary cause of death in such cases [8]. Infection rates for vascular access in dialysis patients with a fistula are 0.5–1.5% per patient-year. This is significantly lower than the infection risk in patients with other vascular approaches. Nevertheless, despite these differences in infection risk among access types, the overall risk of infection remains a significant concern. In our study, infectious complications represented 5.1% of total late complications (twelve cases), with seven managed conservatively and five necessitating surgical intervention. Infections often complicate care due to the associated comorbidities in these patients, such as heart failure and anemia. Infectious processes may lead to local tissue maceration, endangering the anastomosis and risking secondary hemorrhage, or they may spread to organs, with endocarditis or osteoarticular infections documented in 14.5–44% of cases [48].

Venous hypertension affected 16 patients (6.8%), matching literature-reported rates of 6.6–11% [49,50]. This condition is often associated with central venous stenoses and requires prompt intervention, including collateral ligation or conservative management with postural drainage and elastic bandage compression. None of our cases required fistula closure, although distal AVFs may lead to trophic finger lesions.

## 5. Limitations

This study has several limitations that warrant discussion. First, the results of the predictive model reflect only moderate discriminative ability, which may not be sufficient for standalone predictive purposes in clinical decision-making. This might be improved by potentially using additional clinical parameters or advanced methodologies, to enhance its predictive utility and real-world applicability.

Second, the analysis of risk factors in this study could be expanded to address specific influences of demographic, anatomical, and clinical variables. While this study focused on certain key parameters, other potentially influential factors, such as socioeconomic status, environmental exposures, and lifestyle behaviors, were not included in the analysis. These factors could have a significant impact on both disease outcomes and model performance and should be considered in future investigations to provide a more holistic understanding of risk and predictive capabilities. The sample size in this study may limit the generalizability of the findings. Therefore, future studies should aim for larger, more diverse cohorts to ensure their applicability to different clinical settings. Lastly, the study relied on retrospective data, which could introduce biases related to data completeness, quality, or consistency.

## 6. Conclusions

This study shows that complications related to vascular access for hemodialysis are common and can significantly impact patient outcomes. The study revealed that hemorrhage (32.3%) and thrombosis (30.8%) were the most common early complications, with distinct patterns based on demographic and clinical factors. Hypertensive patients had an increased thrombosis risk (33.9%), while diabetes was notably associated with hemorrhage (39.6%) and non-maturation (20.8%). Late complications, predominantly thrombosis (38.6%) and infections (36.7%), were influenced by factors such as age, smoking status, and comorbidities, with hypertension and CVD significantly elevating thrombosis risk. The logistic regression model demonstrated moderate performance in predicting thrombosis, with an accuracy of 64.6%, an ROC AUC of 0.64, a precision of 73%, and a recall of 65.5%. These findings highlight the need for careful patient assessment before and after vascular access creation to reduce the risk of complications. Ensuring regular monitoring of vascular access can help address these challenges. Future research should explore ways to predict and prevent complications, aiming to improve the care and quality of life for patients on hemodialysis.

## Figures and Tables

**Table 1:** Demographic and lifestyle parameters of the study population with early complications.

Parameter	Mean	SD	Percentage (%)
Age	66.2	12.5	-
BMI	26.9	4.5	-
Gender (male)	-	-	62.3
Gender (female)	-	-	37.7
Smoking (never)	-	-	55.4
Smoking (former)	-	-	28.5
Smoking (current)	-	-	16.2

**Table 2:** Univariate analysis of early complications.

Complication	Age (OR, 95% CI)	Gender (OR, 95% CI)	Hypertension (OR, 95% CI)	Diabetes (OR, 95% CI)	CVD (OR, 95% CI)	Smoking (RR, 95% CI)	BMI (OR, 95% CI)
Thrombosis	1.12 (1.05–1.20)	1.25 (1.05–1.50)	1.32 (1.10–1.58)	0.65 (0.53–0.81)	0.90 (0.72–1.12)	1.10 (1.00–1.21)	1.18 (1.05–1.33)
Hemorrhage	1.20 (1.10–1.30)	0.95 (0.80–1.15)	0.57 (0.47–0.69)	1.67 (1.37–2.03)	0.93 (0.71–1.22)	1.04 (0.95–1.14)	1.05 (0.95–1.15)
Edema	1.25 (1.15–1.35)	1.10 (0.90–1.35)	1.47 (1.27–1.71)	0.55 (0.46–0.67)	0.84 (0.68–1.03)	1.48 (1.30–1.68)	1.22 (1.10–1.35)
Non-maturation	1.18 (1.08–1.30)	1.30 (1.10–1.55)	1.10 (0.98–1.24)	1.70 (1.40–2.07)	1.70 (1.35–2.12)	1.76 (1.44–2.16)	1.30 (1.15–1.48)

**Table 3:** Demographic and lifestyle parameters of the study population with late complications.

Parameter	Average	SD	Percentage (%)
Age	62.4	9.6	-
BMI	30.3	6.6	-
Gender (male)	-	-	46.4
Gender (female)	-	-	53.6
Smoking (never)	-	-	49.3
Smoking (former)	-	-	27.8
Smoking (current)	-	-	22.7

**Table 4:** Univariate analysis of late complications.

Complication	Age (OR, 95% CI)	Gender (OR, 95% CI)	Smoking (RR, 95% CI)	Hypertension (OR, 95% CI)	Diabetes (OR, 95% CI)	CVD (OR, 95% CI)	BMI (OR, 95% CI)
Thrombosis	1.03 (1.01–1.05)	1.23 (0.75–2.03)	0.83 (0.76–0.91)	1.42 (1.20–1.68)	1.63 (1.42–1.84)	2.05 (1.85–2.25)	1.02 (0.99–1.05)
Aneurysmal dilatation	1.04 (1.02–1.06)	1.15 (0.88–1.51)	1.21 (1.03–1.41)	1.80 (1.50–2.12)	1.13 (0.94–1.34)	1.20 (1.05–1.36)	1.07 (1.03–1.12)
Infection	1.02 (1.00–1.04)	1.32 (0.90–1.93)	1.12 (1.00–1.26)	0.61 (0.50–0.75)	0.88 (0.75–1.03)	0.93 (0.78–1.10)	1.02 (0.99–1.05)
Vascular steal syndrome	1.04 (1.02–1.06)	1.20 (0.91–1.58)	1.15 (1.00–1.31)	1.34 (1.15–1.56)	1.08 (0.91–1.28)	1.12 (0.96–1.31)	1.09 (1.04–1.13)
Cardiac complication	1.02 (1.00–1.03)	1.30 (0.89–1.88)	1.10 (0.98–1.23)	1.16 (1.00–1.36)	1.10 (0.92–1.31)	1.08 (0.92–1.28)	1.02 (0.99–1.04)
Venous hypertension	1.03 (1.01–1.05)	1.20 (0.89–1.62)	1.11 (0.97–1.26)	1.18 (1.06–1.31)	0.90 (0.78–1.03)	0.94 (0.83–1.07)	1.01 (0.98–1.04)
Posttraumatic hematoma	1.02 (1.00–1.04)	1.10 (0.80–1.48)	1.05 (0.95–1.17)	1.12 (0.97–1.30)	0.92 (0.81–1.05)	0.98 (0.84–1.13)	1.02 (0.99–1.05)

## Data Availability

Data available upon request.
